# 8. False positive mumps serology in a patient with cerebral systemic lupus erythematosus

**DOI:** 10.1093/rap/rky033

**Published:** 2018-09-20

**Authors:** Adnan Hammad, Alasdair Coles, Mark D J Gibson

**Affiliations:** 1 University of Cambridge School of Clinical Medicine, Cambridge University Hospitals, Cambridge, UNITED KINGDOM; 2 Department of Neurosciences, Cambridge University Hospitals, Cambridge, UNITED KINGDOM

**Introduction:** Here we present the case of a 27-year-old SLE sufferer with NPSLE, who had positive serological tests for both CMV and mumps. These were both subsequently confirmed to be false positives by polymerase chain reaction (PCR). While false positive CMV serology in the context of SLE has already been reported in the literature, there has been no report of a similar phenomenon for mumps.

**Case description:** A 27-year-old female was admitted with acute confusion, dysarthria and somnolence. Three days prior to admission she had received a diagnosis of systemic lupus erythematosus in a rheumatology outpatient clinic having been referred with a six month history of arthralgia primarily affecting the knees and small joints of the hands; she had been found to have elevated antibodies against double stranded DNA (dsDNA) and was commenced on prednisone 10 mg daily and hydroxychloroquine 200 mg daily. Over the next three days, she developed progressive confusion and fluctuating somnolence. Upon admission to hospital, examination revealed a GCS of 14 (E4V4M6), equal and reactive pupils, symmetrical facial movements, dysarthric speech, globally brisk tendon reflexes and an extensor plantar response bilaterally. No rashes were noted. Examination of the oral mucosa did not reveal the presence of apthous ulcers. Examination of the joints revealed nil active synovitis. She was found to be anaemic (haemoglobin 93g/L, reference range 120-156g/L), leukopaenic (2.10 x10*9/L, reference range 3.9-10.2 x10*9/L) and lymphopaenic (0.18 x10*9/L, reference range 1.10-4.50 x10*9/L). The platelet count was normal (240 x10*9/L, reference range 150-370 10*9/L). The direct antiglobulin test was positive. She was strongly positive for anti-nuclear antibodies (ANA >1/1000) with a homogenous HEp-2 staining pattern and significantly elevated anti-double stranded DNA titres (>400 IU/ml, reference range 0-10 IU/ml) and had hypocomplementaemia (C3 0.20g/l, reference range 0.75-1.65g/l, C4 0.03, reference range 0.14-0.54g/l). Testing for extractable nuclear antigens was negative (RNP, Sm, Ro, La, Scl, Jo1 & centromere). Clotting studies revealed a prolonged activated partial thromboplastin time (APTT 38 seconds, reference range 25.8-35 seconds) with a normal prothrombin time. MRI imaging of the head revealed multiple areas of T2 hyperintensity, contrast enhancement and restricted diffusion. A CT cerebral angiogram revealed irregularity of the left posterior cerebral artery, distal branches of the left middle cerebral artery pericallosal vessels, findings consistent with an intracranial vasculitis. A CT pulmonary angiogram revealed incidental bilateral segmental pulmonary emboli with resulting pulmonary infarction. Testing for anti-phospholipid antibodies revealed a positive lupus anticoagulant, positive anti-cardiolipin IgM (47 mplu/ml, reference range 0-9 mplu/ml) and IgG (12 gplu/ml, reference range 0-9 gplu/ml) and positive beta-2-glycoprotein-1 IgM (10 u/ml, reference range 0-6 u/ml) and IgG (8 u/ml, reference range 0-6 u/ml). Serological testing was negative for Borrelia burgdoferi IgG, Aspergillus galactomannan antigen, Epstein-Barr virus IgG and IgM, hepatitis B core antibody, hepatitis B surface antigen, HIV antibody, HIV antigen, toxoplasma IgG, and Treponemal antibody. CSF analysis showed a normal opening pressure (16cmH2O, reference range 0-20cmH2O), ^elevated^ protein (1.04g) lymphocytosis (66 lymphocytes, reference range 0-5), decreased glucose (CSF glucose 1.3mmoml/L, serum glucose 4.7 mmol/L) mildly increased neutrophil count (8, reference range 0-5), and a red cell count 48. No organisms were seen on a Gram stain or Ziehl-Neelsen stain, viral PCR was negative for herpes zoster, varicella zoster and adenovirus and CSF culture, including mycobacterial culture, revealed no growth. CSF cytology revealed lymphocytosis with some evidence of lymphophagocytosis. A provisional diagnosis of active systemic lupus (SLEDAI-2K score 44) with cerebral lupus with secondary CNS vasculitis and probable anti-phospholipid syndrome.

Upon presentation empirical ceftriaxone and acyclovir were commenced to cover for a meningoencephalitis. These were discontinued following CSF analysis. Upon making the diagnosis of active lupus therapeutic low molecular weight heparin (tLMWH), pulsed intravenous methylprednisolone (IVMP) therapy and plasma exchange were commenced. Five consecutive plasma exchanges were performed with normal saline and human albumin solution used as the replacement fluid. Fresh frozen plasma was used instead of human albumin solution for the third exchange because of transient hypofibrinogenaemia (1.07 g/L, reference range 1.46-3.33 g/L. The patient’s clinical picture remained unchanged. It was decided to escalate therapy to pulsed intravenous cyclophosphamide (CYC), however this was delayed by three days because of concerns of an alternative diagnosis of a viral encephalitis, because she had positive serology for cytomegalovirus (CMV) IgM & IgG and Mumps IgM & IgG. Confirmation of infection was sought through PCR of blood and CSF. In house testing for CMV PCR in the blood was negative. Mumps PCR tests were delayed because these are done at an external laboratory. It was decided that treatment with cyclophosphamide should start even without this test result, because it emerged that she had received the mumps, measles and rubella (MMR) vaccine in 1990 and the clinical picture was felt not to be in keeping with a mumps encephalitis. She had intravenous pulsed cyclophosphamide therapy as per the CYCLOPS regimen. Subsequent mumps PCR was negative in both salivary and cerebrospinal fluid samples. Pulsed cyclophosphamide and oral steroids were continued. The clinical picture improved with improving neurological function and improved MRI appearances. The positive mumps IgM and CMV IgM serological results were attributed to false positives in the context of active systemic lupus erythematosus with positive anti-phospholipid antibodies.The clinical picture gradually improved over the following two weeks with improving neurological function and resolving MRI appearances. The positive mumps IgM and CMV IgM serological results were attributed to false positives in the context of active systemic lupus erythematosus with positive antiphospholipid antibodies. The patient’s care was transferred from our tertiary unit to her local hospital where she completed pulsed cyclophosphamide as an outpatient followed by the introduction of mycophenolate mofetil. She was lost to further follow up at this hospital.

**Discussion:** The interplay between autoimmune diseases and infections is a complex one, and the serological diagnosis of infections in individuals suffering from autoimmune diseases—especially lupus—is not straightforward, owing to the possibility of false positive results on serological tests. This has been well reported for a number of infectious diseases, such as HIV, syphilis, toxoplasmosis and CMV, in the context of SLE. In this report, we described a young patient who had positive serology for both CMV and mumps. Subsequent negative PCR results confirmed that these were false positives. While false positive CMV serology has already been reported in the literature, there has been no reports of false negative mumps serology. False positive CMV serological results have been reported in the context of SLE, both in pregnant women and outside pregnancy. Similarly, false positive serology for syphilis in SLE has been recognised for a long time, having been first reported back in the 1950’s. Others have reported similar findings since then. False positive HIV serology has also been reported in SLE patients, while false positive Lyme disease serology in SLE patients is not uncommon, with one study reporting incidence as high as 40%. The mechanisms underlying false positive serology in these different contexts have not been fully elucidated, but it is likely that a common mechanism underlies this phenomenon. The most likely mechanism involves dysregulated B lymphocyte proliferation and differentiation, which is likely to result in the production of a diverse range of anti-viral and anti-bacterial antibodies in addition to non-specific sticky immunoglobulins that give high backgrounds on serological assays and are therefore misdiagnosed as positive. Two major types of tests for syphilis are commonly used; treponemal and non-treponemal. The non-treponemal tests detect anti-treponemal antibodies using a cardiolipin/lecithin/cholesterol mixture. As a consequence of the nature of the antigens that these tests utilise, they are associated with a relatively high rate of false positives in autoimmune conditions such as lupus. This has been explained by the observation that anti-dsDNA and anti-single stranded DNA (anti-ssDNA) antibodies are able to cross-react with phospholipids, including cardiolipin. While treponemal tests are thought to have higher specificities than non-treponemal tests, false positive results have been reported for treponemal tests in the context of SLE as well. For example, serum obtained from SLE patients tested using the fluorescent treponemal antibdy-absorption (FTA-ABS) test was found to result in a beaded appearance, in contrast with the more homogeneous appearance expected in someone truly positive for syphilis. This beaded pattern was therefore recognised as being indicative of a false positive result. It is likely that this atypical pattern on FTA-ABS was the result of cross-reactive autoantibodies in the SLE patients’ serum. There is evidence for polyclonal B cell activation underlying false positive serology for HIV in SLE as well. It was observed that antibodies cross-reactive to HIV p24 gag protein could be found in SLE patients’ serum. Indeed, antibodies directed against HLA-DR molecules (an MHC class II receptor) have been reported to be present in SLE patients’ serum, and there is significant sequence homology between MHC class II receptors and HIV antigens, which may explain the aforementioned cross-reactivity. Similarly, the false positive results on Borrelia ELISAs have been suggested to be due to either the non-specific binding of SLE immune complexes to microtitre wells, or the non-specific cross-reactivity of autoantibodies found in SLE to Borrelia antigens (either cytoplasmic or nuclear, or both) in microtitre wells. Alternative explanations have been proposed, which speculate on the presence of other infections that may result in the production of cross-reactive antibodies. For example, false positive CMV serology in the context of SLE has been postulated to be due to the presence of another infection, e.g. Epstein-Barr virus (EBV), which might be able to result in a polyclonal B cell reaction. Thus, anti-CMV antibodies may be produced, thereby giving false positive reactions. Antibodies directed against other herpes viruses also appear to show significant cross-reactivity, which could potentially explain CMV false positive serology in individuals suffering from other herpes infections. Similarly, false positive HIV serology in SLE patients has been proposed to be linked to a possible role for retroviruses in the pathogenesis of SLE. HIV is considered unlikely to be the retrovirus in question but, due to significant homology between retroviruses, it is likely that an SLE sufferer who might have an associated retroviral infection would possess antibodies against the aforementioned retrovirus that would also be cross-reactive to HIV antigens. To conclude, there was a delay in this patient receiving immunotherapy for her life-threatening cerebral lupus because of a failure to recognise that positive serology results for both CMV and mumps might be false positive, as subsequently confirmed when PCR analysis was performed. False positive CMV serology has been previously reported in SLE, and the possible mechanisms underlying this phenomenon were discussed above. False positive mumps seroreactivity in SLE patients, on the other hand, has so far not been reported in the literature. However, given the non-specific polyclonal nature of the B lymphocyte response induced by SLE and the diversity of autoantibodies found in SLE, it is possible that SLE patients may possess a number of antibodies capable of cross-reacting with mumps antigens, thereby resulting in false positive results on mumps serology tests. It is important for clinicians to recognise the possibility of false viral serology in people with lupus to avoid unnecessary delays in administering immunotherapy.

**Key Learning Points:** Numerous serological assays against infectious diseases have been associated with false positive results in lupus patients. Positive serological tests for infections in patients with active lupus should be interpreted within the clinical context and should not in themselves preclude commencing immunotherapy for active lupus. There is a role for PCR in determining true and false seropositive patients.

**Disclosure: A. Hammad:** None. **A. Coles:** None. **M.D.J. Gibson:** None.

